# Histone acetylases are required for iron homeostasis in yeast: Histone acetylases and iron homeostasis in yeast

**DOI:** 10.3724/abbs.2025040

**Published:** 2025-03-17

**Authors:** Jian Zhang, Yong Xue, Xinya Zhang, Renjie Qi, Yaqi Zhang, Chen Lu, Zhidan Luo

**Affiliations:** 1 Jiangsu Key Laboratory of Marine Biological Resources and Environment Jiangsu Key Laboratory of Marine Pharmaceutical Compound Screening Jiangsu Ocean University Lianyungang 222005 China; 2 Co-Innovation Center of Jiangsu Marine Bio-industry Technology Jiangsu Ocean University Lianyungang 222005 China; 3 Jiangsu Institute of Marine Resources Development Lianyungang 222005 China

Iron, an ancient and essential transition metal, is involved in various biological functions, including oxygen transport, DNA synthesis, heme production, and iron-sulfur clusters, which participate in electron transport, DNA repair, and other cellular processes. However, excessive iron can lead to oxidative stress, lipid peroxidation, and cell damage. Thus, maintaining the iron content within an appropriate safe range and maintaining the balance of iron metabolism play crucial roles in both cellular function and human health [1].

An important aspect of maintaining the balance of iron homeostasis is the regulation of the iron uptake system. In *Saccharomyces cerevisiae*, cells can either obtain iron from the external environment via the non-reducing siderophore transport system or transport iron from the extracellular space to the intracellular space via the reducing iron transport system [2]. Iron uptake system-related genes are regulated mainly by the transcription factor Aft1p. During iron deficiency, Aft1p translocates into the nucleus, binds to genes involved in iron metabolism, and regulates the expressions of genes involved in iron uptake systems [3]. In addition, when there is a problem in the synthesis of iron-sulfur clusters in the mitochondria, such as the lack of the iron chaperone Yfh1p, which promotes the synthesis of iron-sulfur clusters, the transcription and nuclear entry of the transcription factor Aft1p are also activated, thereby regulating the expressions of iron metabolism-related genes [4].

In addition to transcription factors, gene expression is also regulated by histones and their modifications at the epigenetic level. For example, histone H3K4 methylation is related to gene activation, H3K36 methylation plays an important role in the elongation of transcription, and histone acetylation results in the loss of nucleosome structure and facilitates gene expression [5]. Therefore, histone modifications should also play important roles in the regulation of iron homeostasis. The relationship between histone modifications and iron homeostasis has been reported in the literature. For example, the DNA methylation-binding protein MBD5 can change histone acetylation in the promoter region of the ferritin gene by recruiting the histone acetylase KAT2A protein [6]. Histone acetylation has also been reported to be reduced in iron-deficient environments [ 7, 8] , and direct effects of histone acetylation on iron homeostasis gene loci have also been reported both in *C*. *albicans* [9] and mammals [10]. More recently, the histone H3-H4 tetramer was found to be a copper reductase enzyme, and H3-mediated Cu ^+^ toxicity is a major determinant of the cellular functional pool of iron-sulfur (Fe-S) clusters [ 11, 12] . However, information on the role of histone modifications in the regulation of iron homeostasis is limited. The mechanism by which and how histone modifications are involved in the transcriptional regulation of iron uptake-related genes or the iron deficiency response require further investigation.

To determine whether histone acetylation and methylation are involved in the iron deficiency response, we first deleted the histone acetylase genes, including *GCN5* (histones H2B and H3 N-terminal lysine acetylase, partial deletion of the *ADA2* interaction sequence [13]), *RTT109* (H3 lysine 9 and 56 acetylase), *SAS2* (H4 lysine 16 acetylase), and *YNG2* (subunit of the histone acetyltransferase complex NuA4 for acetylation of histone H4 or histone H2A) in the wild-type (WT) strain, as well as the histone methyltransferases *SET1* (H3 lysine 4 methyltransferase), *SET2* (H3 lysine 36 methyltransferase) and *DOT1* (H3 lysine 79 methyltransferase). The genomic deletions were confirmed by colony PCR and genomic coverage analysis, as depicted in Supplementary Figures S1 and S2. The sensitivity of single histone modification enzyme mutants to iron deficiency induced by the iron chelator bathophenanthroline disulfonate (BPS) was tested. As shown in Supplementary Figure S3, the histone acetylation-related mutants *gcn5 _1–316_*, *rtt109Δ*, *sas2Δ*, and *yng2Δ* did not exhibit significant growth defects compared with the WT on the YPD + BPS plate. None of the histone methyltransferase knockout strains presented significant growth defects. As a positive control, the iron-responsive transcription factor gene *AFT1* knockout strain grew slowly on YPD + BPS plates.

It is possible that histone modifications do not have a strong effect on the equilibrium status of iron deficiency but still regulate transcription induction during the iron deficiency response. To investigate the role of histone modifications during the induction of the iron deficiency response, the expressions of iron response genes in the wild-type and mutant strains before and 4 h after BPS treatment was measured via RNA-Seq. As shown in Figure 1A, analysis of all the iron homeostasis genes (annotated in the Saccharomyces Genome Database, 28 genes) revealed that the difference in the median expression level of iron homeostasis genes before and after BPS treatment was significantly greater in the *gcn5 _1–316_* and *sas2Δ* strains than in the BPS-treated WT cells or other mutants. The genes were further checked individually via a heatmap and a bar graph ( Figure 1B,C). Interestingly, the activation of the multicopper ferroxidase gene *FET3* after BPS treatment, which imports iron when it is present at low concentrations for iron uptake, was further enhanced in *gcn5 _1–316_* and *sas2Δ* but similar in *rtt109Δ* and methyltransferase knockout strains *set1Δ*, *set2Δ* and *dot1Δ* compared with WT. Notably, the expression of FET3 before BPS treatment was greater *in yng2Δ* but lower in *gcn5 _1–316_* than in WT. FIT2, which is involved in the uptake of siderophore-bound iron from the environment, was highly activated in *gcn5 _1–316_* and *set1Δ* compared with WT or other mutants ( Figure 1C). CTH2 or TIS11, a conserved mRNA-binding protein that modulates the metabolic response to iron deficiency, was also specifically activated in *sas2Δ*, *gcn5 _1–316_* and *yng2Δ* strains ( Figure 1C), and the induction of *CTH2* was not obvious in the WT and methyltransferase knockout strains (these genes were not fully activated in the current BPS treatment). The alterations in the expressions of the *CTH2*, *FET3*, and *FIT2* genes under iron-deficient conditions were also confirmed through a combined analysis of RNA-sequencing and reverse transcription polymerase chain reaction (RT‒PCR) data ( Supplementary Figure S4). The induction of *FET3*, *FIT2*, and *CTH2* in WT and mutants is also shown in the genome browser ( Figure 1D). Taken together, our transcriptome analyses revealed that, compared with other histone modification enzymes, the loss of histones acetylates Gcn5p and Sas2p has a stronger effect on the induction of iron homeostasis genes under iron deficiency conditions induced by BPS.

**Figure 1:**
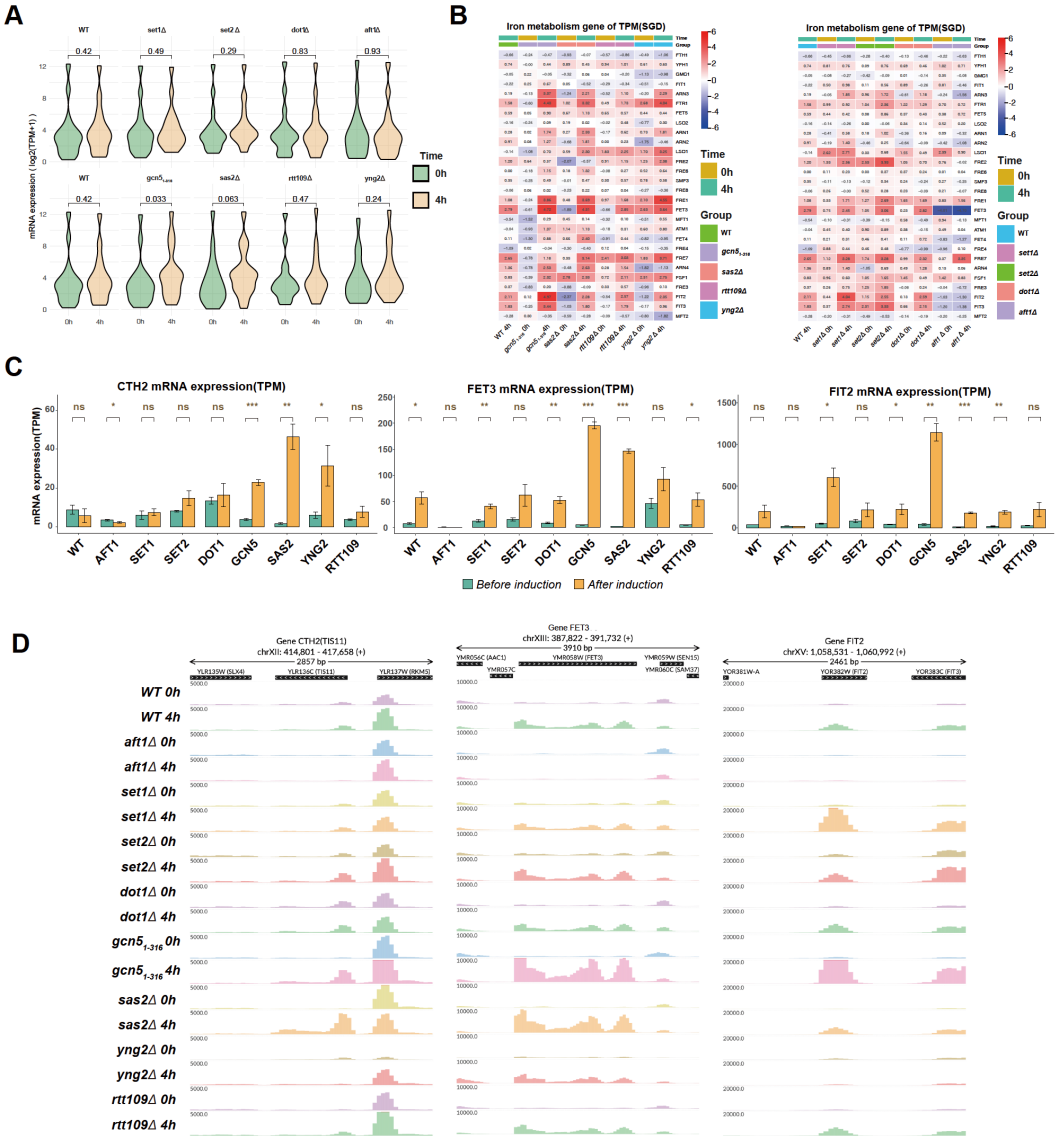
Figure 1 Effects of the loss of histone methylation or acetylation enzymes on the induction of iron metabolism genes (A) Violin plots of the expressions of 28 iron homeostasis-related genes (log2 TPM +1) in the WT, aft1Δ,set1Δ,set2Δ,dot1Δ,gcn5 1–316 ,sas2Δ,yng2Δ, and rtt109Δ strains before and after BPS treatment. (B) Heatmap of the relative expressions of 28 iron homeostasis-related genes in the above strains. The log2 ratio of mRNA expression in all strains versus WT (before BPS treatment) was used in the heatmap. (C) Bar charts with P values showing the expression level (TPM) of the CTH2,FET3, and FIT2 genes before and after BPS treatment in the mutated strains. *P < 0.05, **P < 0.01, ***P < 0.01; and ns, not significant, after induction compared with before induction. (D) Genome browser of the CTH2,FET3, and FIT2 genes before and after BPS treatment in the mutated strains. Violin plots and bar plots with the Mann-Whitney U test were used to determine the significance of (A,C).

Deficiencies in the biosynthesis of Fe-S clusters in mitochondria, such as the loss of the mitochondrial iron chaperone Yfh1p, also induce the iron deficiency response. Therefore, we next investigated whether histone acetylation is also involved in the regulation of iron stress induced by YFH1 inhibition. We replaced the promoter *of YFH1* by the *GAL1* promoter ( *pGAL-YFH1*) to enable inducible YFH1 expression and analysis of genetic interactions. Growth in the presence of glucose, which represses the expression of YFH1, resulted in a growth defect but not in the presence of galactose. The growth effect of a lack of histone modification enzymes with or without the inhibition of YFH1 expression was tested on YPD and YPG plates. Interestingly, mutation of *gcn5 _1–316_* caused a clear recovery effect on cell growth via the repression of YFH1. Knockout of *SAS2* had a weak effect on recovery when SAS2 was combined with *YFH1* repression ( Figure 2). However, the growth defect is much more severe when *rtt109Δ* is combined with *YFH1* shutoff. Knockout of *YNG2* did not differ when YNG2 was combined with *YFH1* repression. These findings suggest that histone acetylation and working biosynthesis of the iron-sulfur system function complementarily to ensure optimal cell growth. Among these genes, *Gcn5p* and *Rtt109p* play specific roles in the cellular response to iron deficiency.

**Figure 2:**
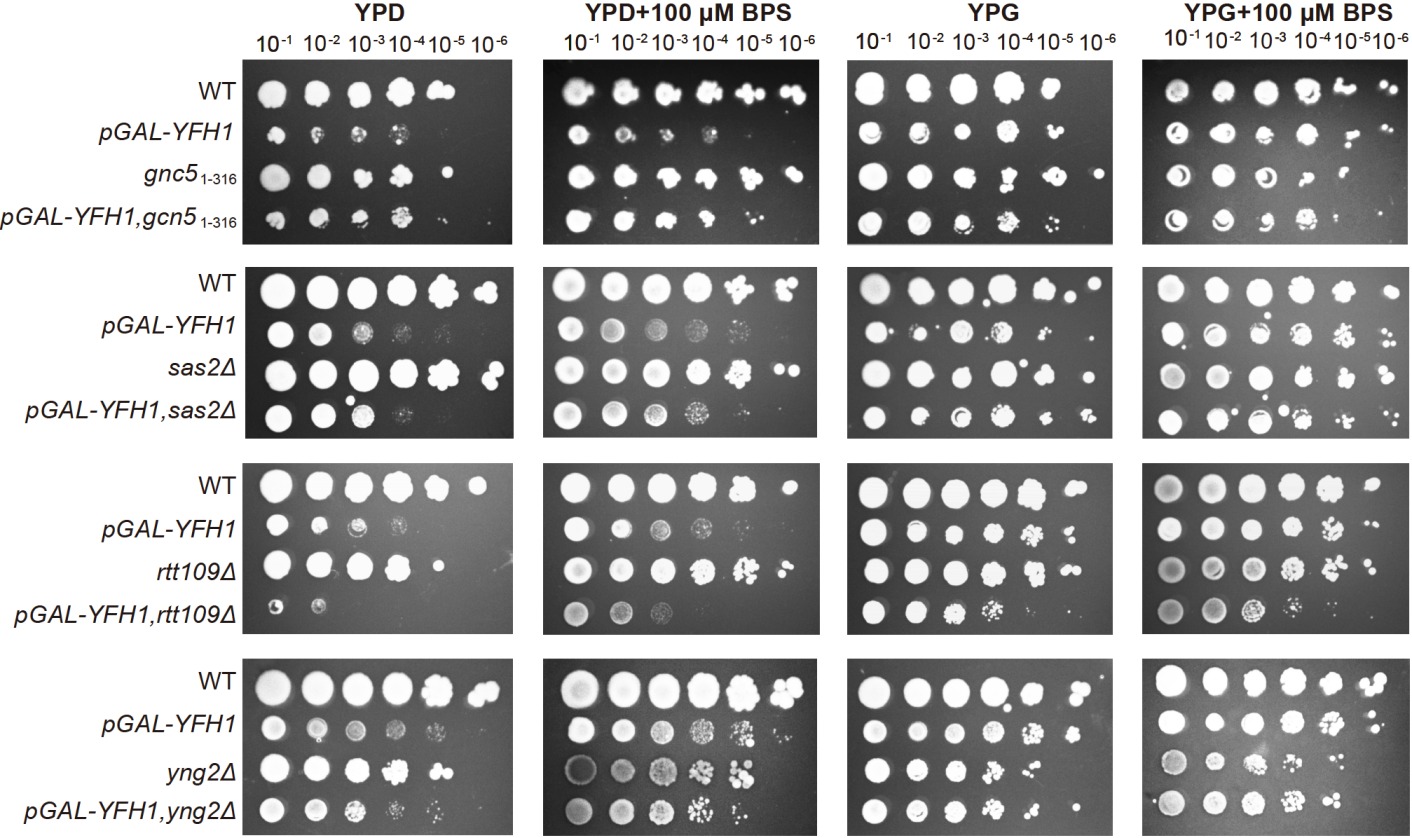
Figure 2 The growth effect of histone acetylation induced by *YFH1* inhibition Serial 10-fold dilutions of yeast strains WT or acetylation mutants with inducible YFH1 (pGAL-YFH1) were grown on YPD or YPG plates (with or without a BPS) at 30°C. The plates shown here were incubated for 3 days. YFH1 expression was inhibited in the YPD plate.

In summary, we investigated the role of individual histone modification enzymes during the response to iron deficiency and revealed that the histone acetylases Gcn5p and Sas2p play specific roles in gene activation during the iron deficiency response induced by BPS and that knockout of *GCN5* or *SAS2* has a recovery effect when combined with YFH1 repression. Considering the lower histone acetylation level in iron-deficient environments [ 7, 8] , acetylation of histones by Gcn5p or Sas2p may inhibit the induction of iron uptake genes in iron-deficient conditions induced by BPS, thereby lowering iron uptake. The SAGA histone-modifying complex has also been found to function as a molecular switch to fine-tune the tight control of iron homeostasis gene expression in *C*. *albicans* [9]. The difference in growth effects and gene induction between Gcn5p and Sas2p may be due to the different roles of histone H3 and H4K16 acetylation in the distinct iron induction mechanisms of BPS and *YFH1* repression. A study in mammals demonstrated that histone deacetylase 3 and its cofactor NCOR1 regulate hepcidin gene expression, thereby influencing cellular iron deficiency [10]. However, whether Gcn5p or Sas2p directly or indirectly regulates these iron uptake genes and their differentiated functions in different iron deficiency responses require further investigation. We also found that Rtt109p is uniquely important in iron-sulfur cluster deficiency, possibly because Rtt109p promotes genome stability or H3-mediated Cu ^+^ toxicity [12] but not gene transcription regulation. In conclusion, our study demonstrated the distinct roles of different histone modifications in the regulation of iron metabolism.

## Supporting information

503FigS1-S4

## Supplementary Data

Supplementary data is available at *Acta Biochimica et Biophysica Sinica* online.
