# Supplementary material for: Histone acetylases are required for iron homeostasis in yeast: Histone acetylases and iron homeostasis in yeast
Source: Acta Biochim Biophys Sin (Shanghai). 2025 Mar 17;57(6):1029–32. doi: 10.3724/abbs.2025040 (PMC12247131; doi:10.3724/abbs.2025040)
Supplement: 503FigS1-S4 [file 503FigS1-S4.docx]

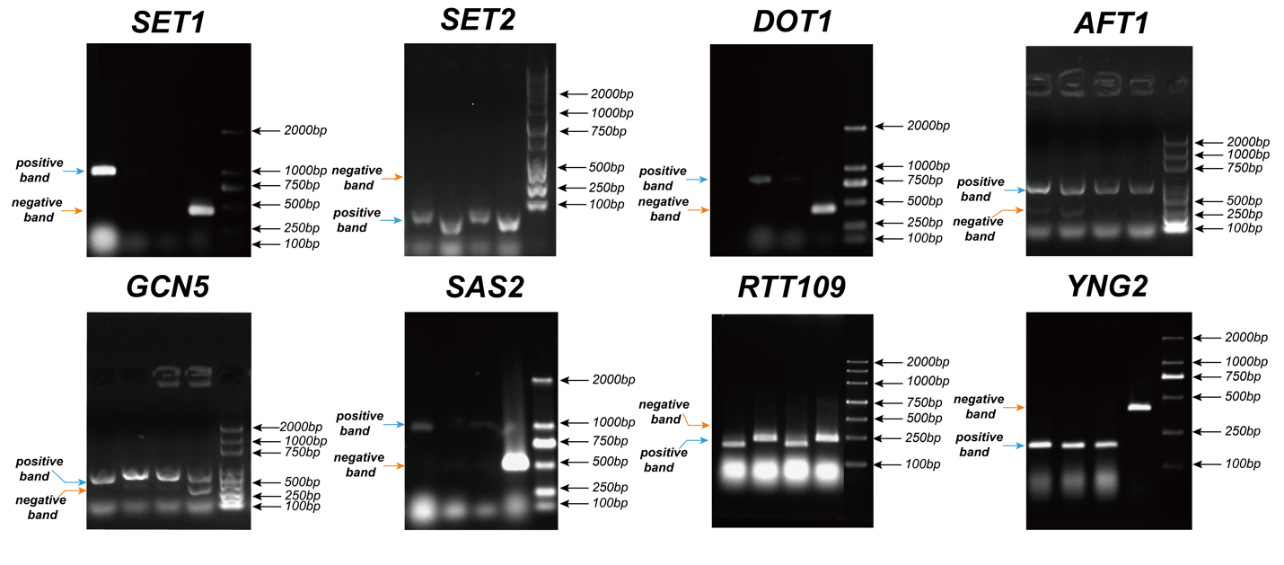


**Supplementary Figure S1. Verification of the histone modification enzyme knockout efficiency via colony PCR** Positive band represents a successful gene knockout, characterized by the presence of a distinct band at the expected molecular weight on the agarose gel; negative band represents a failed gene knockout attempt, indicated by the absence of the expected band or the presence of a band at an unexpected molecular weight.

**
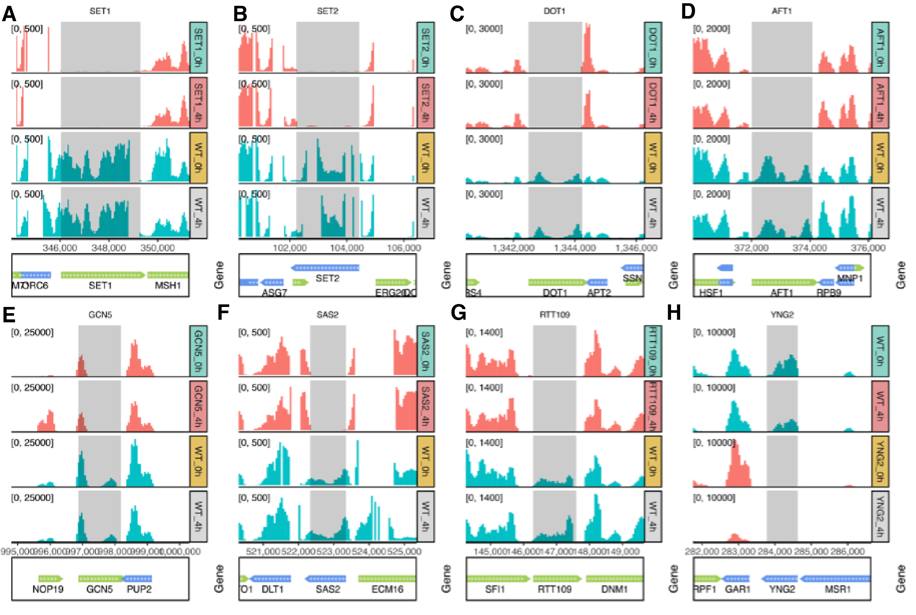
**

**Supplementary Figure S2. Genomic coverage of genes knocked out via RNA sequencing** (A-H) RNA sequencing genome browser plots for the genes *SET1*, *SET2*, *DOT1*, *AFT1*, *GCN5*, *SAS2*, *RTT109*, and *YNG2*, respectively. Each plot illustrates the genomic coverage, with the gray regions indicating the positions of the targeted gene knockouts in the genome. The absence of an RNA signal in these gray areas confirms the successful knockout of the corresponding genes.


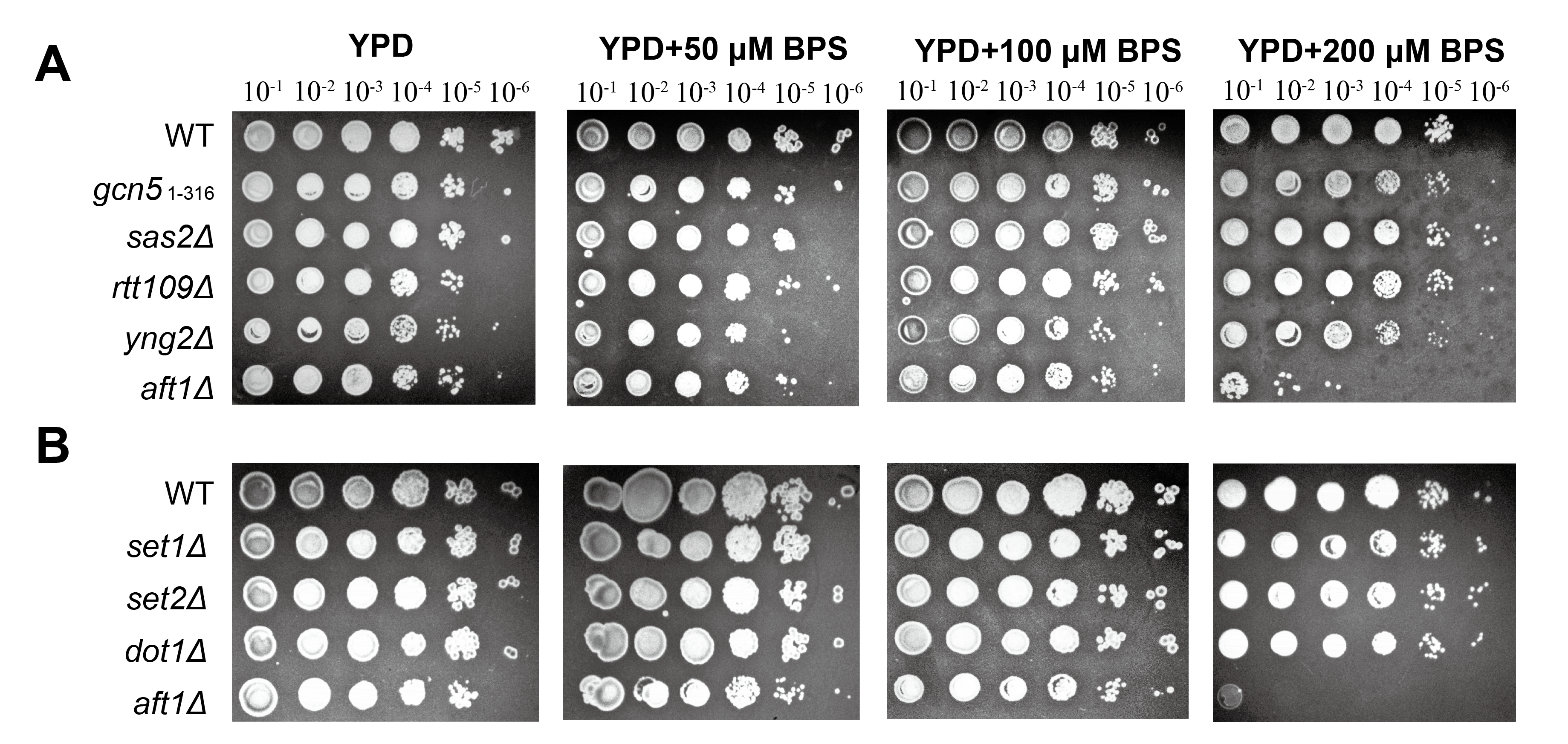


**Supplementary Figure S3. Effect of single histone acetylation or methylation mutation on BPS sensitivity** (A) Serial 10-fold dilutions of yeast strains WT or histone acetylation single mutants were grown on YPD or YPD plates supplemented with varying concentrations of BPS at 30°C. The plates shown here were incubated for 3 days. (B) Same as A but for histone methylation single mutants.


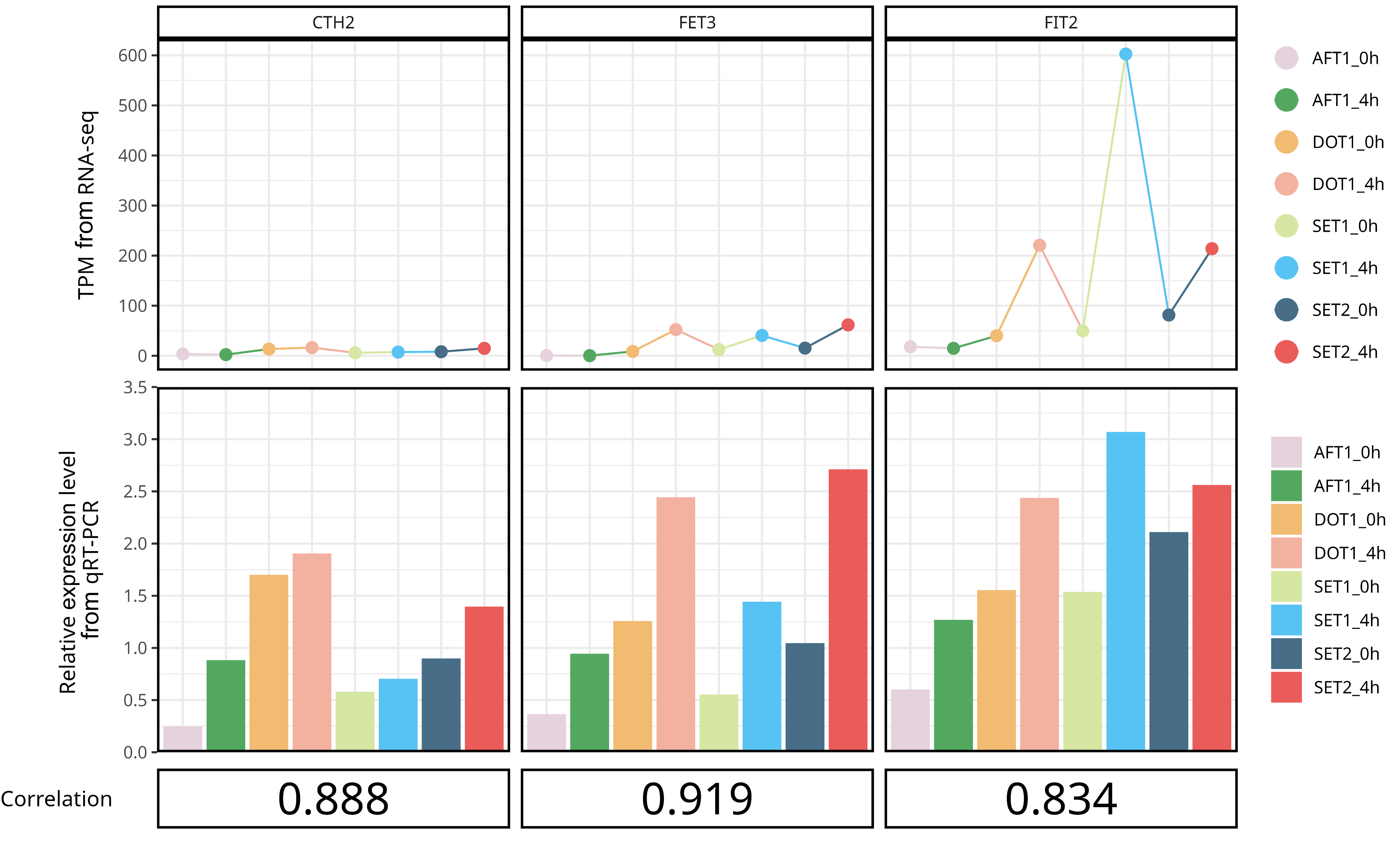


**Supplementary Figure S4. Correlation analysis between RT‒qPCR expression levels and RNA-seq TPM data** This figure presents the correlation analysis between the relative expression levels determined by RT-qPCR and the transcripts per million (TPM) values obtained from RNA sequencing. The correlation coefficient was calculated via the Pearson method, which measures the linear correlation between two datasets.
